# Case report: Persistent syndrome of inappropriate antidiuresis after traumatic brain injury: spontaneous resolution and impact on RAAS and bone metabolism over five years

**DOI:** 10.3389/fendo.2024.1509060

**Published:** 2025-01-24

**Authors:** Yaoxia Liu, Jiao Tang, Mingwei Zhou, Haotian Huang, Tao Wang, Min Zhang

**Affiliations:** ^1^ Department of Geriatric Endocrinology, Sichuan Provincial People’s Hospital, School of Medicine, University of Electronic Science and Technology of China, Chengdu, China; ^2^ Department of Pediatrics, West China Second University Hospital, Sichuan University, Chengdu, China; ^3^ Key Laboratory of Birth Defects and Related Diseases of Women and Children (Sichuan University), Ministry of Education, Chengdu, China; ^4^ Department of Pediatrics, The First People’s Hospital of Longquanyi District, Chengdu, China; ^5^ School of Medicine, University of Electronic Science and Technology of China, Chengdu, China

**Keywords:** SIAD, hyponatremia, traumatic brain injury (TBI), tolvaptan, renin-angiotensin-aldosterone system (RAAS), bone metabolism

## Abstract

The syndrome of Inappropriate Antidiuresis (SIAD) is a well-known cause of hyponatremia and can be associated with various etiologies, including traumatic brain injury (TBI). Most cases of SIAD following TBI exhibit a pattern in which hyponatremia develops several days to weeks after the trauma and resolves within a few weeks. Here, we present a rare case of persistent SIAD caused by TBI that resolved spontaneously after five years. The patient experienced prolonged hyponatremia for several years and was ultimately diagnosed with post-traumatic SIAD after excluding other potential causes. Notably, the patient exhibited an unusual sensitivity to tolvaptan, accompanied by decreased renin levels and increased bone turnover markers. The condition resolved spontaneously after five years, with renin, aldosterone, and bone turnover markers returning to normal upon re-evaluation.

## Introduction

The syndrome of inappropriate antidiuresis (SIAD) is the most common cause of hyponatremia in clinical practice (1, 2). SIAD is characterized by euvolemic hyponatremia, inappropriate urinary concentration, and reduced free water excretion, primarily due to elevated plasma vasopressin (AVP) levels (1). Its clinical manifestations are non-specific, varying according to both serum sodium concentration and the rate of its decline. Moderate symptoms include nausea, confusion, and headache, while severe cases can progress to cardiorespiratory distress, profound somnolence, seizures, and coma (3). The key diagnostic criteria for SIAD include plasma hypo-osmolality (<275 mOsm/kg), inappropriate urine concentration (urine osmolality >100 mOsm/kg), urine sodium >30 mmol/L, clinical euvolemia, and the exclusion of hypothyroidism or adrenal insufficiency (1, 3).

SIAD has been associated with a variety of etiologies, most commonly in conjunction with pulmonary malignancies, surgery, medications, and central nervous system (CNS) disturbances. Traumatic brain injury (TBI) accounts for approximately 2.5% of SIAD cases (1). Hyponatremia secondary to SIAD is a recognized complication of TBI, usually mild and transient, developing days to weeks post-trauma and typically resolving spontaneously within a few weeks (1).

This report presents a rare case of spontaneous resolution of SIAD caused by TBI, persisting for five years. The patient experienced prolonged hyponatremia and, after ruling out other causes, was diagnosed with TBI-induced SIAD. During the illness, the patient exhibited unusual sensitivity to tolvaptan, along with decreased renin, elevated aldosterone levels, and increased bone turnover markers. After five years, the condition resolved spontaneously, with normalization of the renin-angiotensin-aldosterone system (RAAS) and bone metabolic markers.

## Case presentation

On August 12, 2017, a 29-year-old female patient sustained a traumatic brain injury after falling from a bicycle, landing on the posterior right side of her head, which led to an altered level of consciousness. Emergency CT imaging revealed an acute subdural hematoma in the left frontal, temporal, and parietal regions, causing significant compression of the adjacent brain parenchyma, a midline shift to the right, and the formation of brain herniation. Additionally, traumatic subarachnoid hemorrhage, brainstem blurring, and substantial cerebral edema were observed. A scalp hematoma was noted over the right parietal area. One hour after the trauma, the patient underwent an emergency craniotomy for hematoma evacuation and decompressive craniectomy under general anesthesia. Her preoperative serum sodium was 133 mmol/L (reference range: 137-147 mmol/L), but intraoperative monitoring revealed a drop to 127 mmol/L. Postoperatively, she was transferred to the intensive care unit (ICU), where intravenous sodium supplementation and other treatments were administered to maintain her serum sodium levels between 136-141 mmol/L. Gradually, the patient regained consciousness and was transferred to the general ward two weeks later. During this time, she had normal blood pressure but suffered from constipation, abdominal distension, fatigue, and memory impairment. Her serum sodium was found to be 120.7 mmol/L, which improved with sodium supplementation. She was discharged with limited movement in the second and third toes of her right foot.

**Figure 1:**
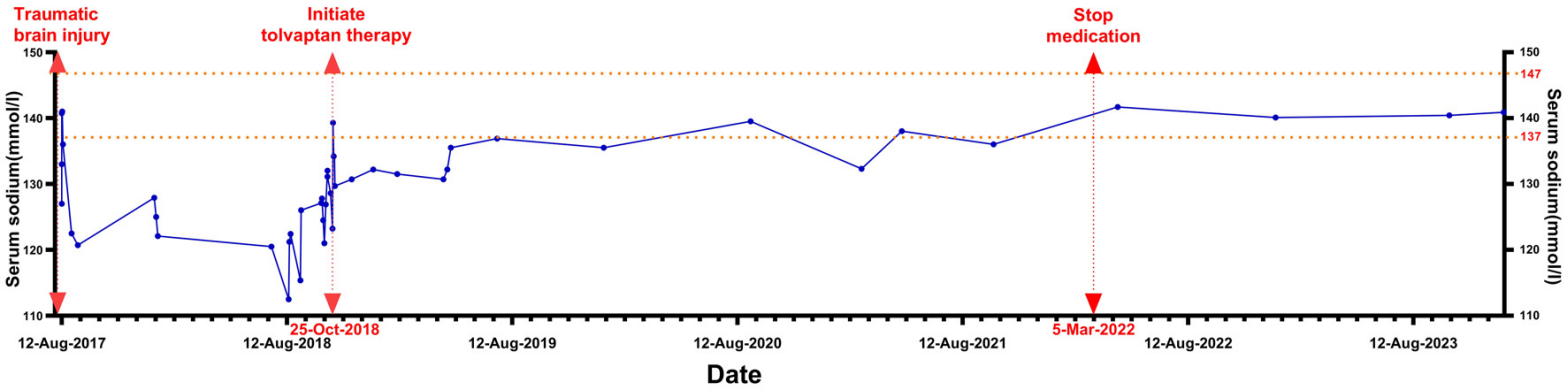
The curve of the patient's serum sodium changes.

In the months following discharge, the patient experienced recurrent episodes of nausea and weakness. Her serum sodium levels fluctuated between 112.5-127.9 mmol/L (**Figure 1**), while chloride levels ranged from 88.4-93.8 mmol/L (reference range: 99-110 mmol/L). Blood potassium and blood pressure remained normal, and her sodium levels improved with both intravenous and oral supplementation; however, they consistently remained below normal.

**Figure 2:**
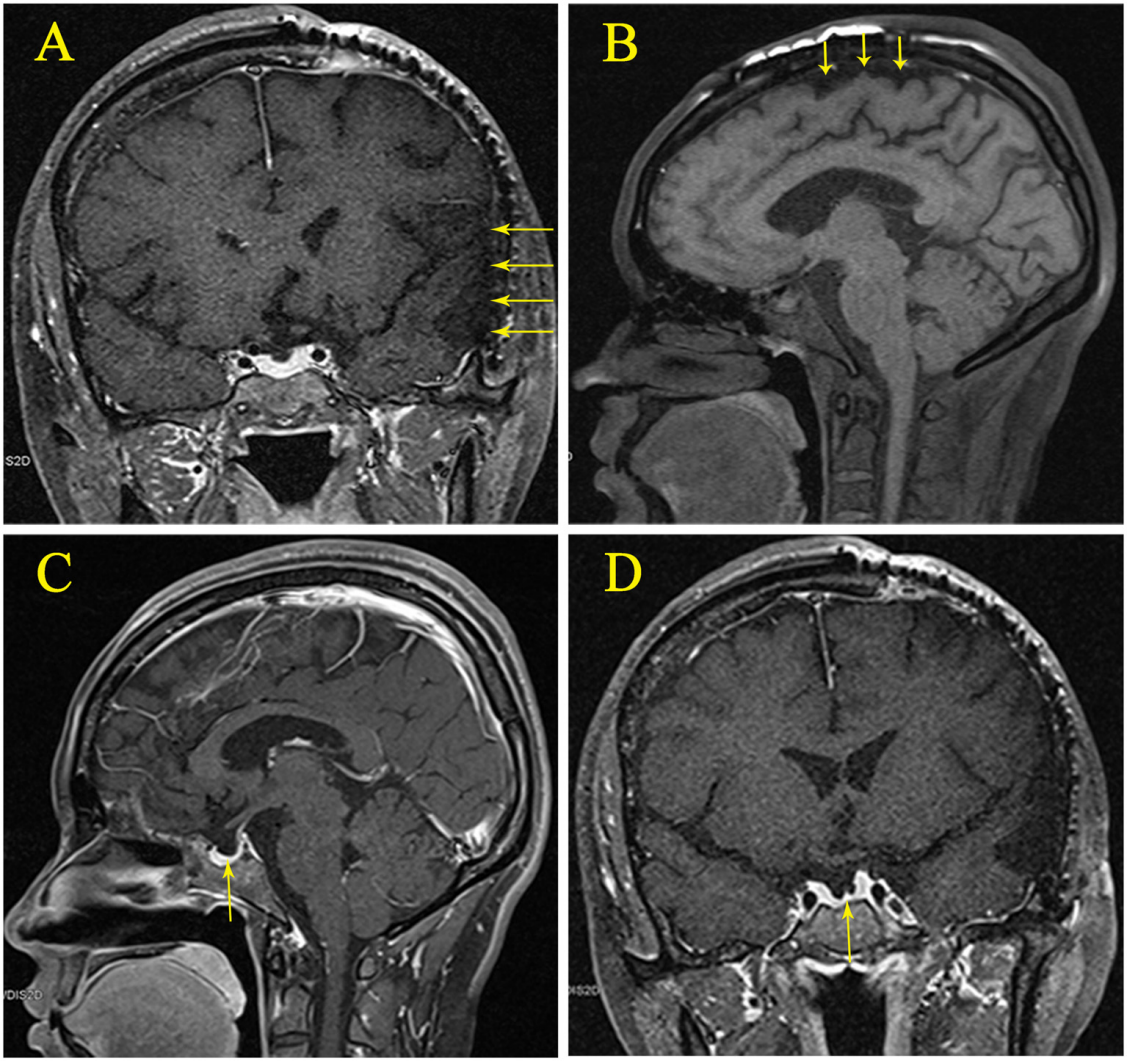
**(A)** Magnetic resonance imaging (MRI) showing post-traumatic encephalomalacia in the left parietal, temporal, and insular lobes. **(B)** MRI showing post-traumatic encephalomalacia in the parietal lobe. **(C)** Sagittal view of an empty sella with the pituitary gland. **(D)** Coronal view of an empty sella with the pituitary gland.

On October 7, 2018 (14 months post-trauma), the patient was readmitted for hyponatremia. A review of the patient’s history revealed no medication use that could have caused hyponatremia. Eight years prior, she had a full-term, normal delivery of a male infant, with normal serum sodium levels at the time of delivery. Menstrual cycles are regular. Physical examination: Temperature 36.1°C, Pulse 60 beats/min, Respiration 20 breaths/min, Blood Pressure 126/90 mmHg. The patient was alert, with visible surgical scars on her head. There was limited movement in the second and third toes of the right foot, accompanied by decreased pain and touch sensation. Pathological signs in the limbs were negative. The MRI showed encephalomalacia in the frontal, parietal, temporal, and insular lobes due to traumatic brain injury. An empty sella was observed, but no abnormal signals or abnormal enhancement were detected in the pituitary gland (**Figure 2**).

Serum sodium levels were measured multiple times, ranging from 121-126.9 mmol/L (**Figure 1**), with chloride levels between 87 and 93.8 mmol/L. Serum osmolality was assessed twice, showing values of 251 and 271 mOsm/kg H₂O (reference range: 275-305 mOsm/kg H₂O). Urine osmolality readings were 590 and 906 mOsm/kg H₂O (reference range: 50-1200 mOsm/kg H₂O), with urine sodium concentrations were 171.7 and 182.7 mmol/L, respectively. Complete blood count indicated hemoglobin of 105 g/L (reference range: 115-150 g/L) and hematocrit of 0.31 L/L (reference range: 0.35-0.45 L/L). Biochemistry results included albumin at 46.0 g/L (reference range: 40.0-55.0 g/L), urea at 2.40 mmol/L (reference range: 2.95-7.70 mmol/L), and uric acid at 114.0 µmol/L (reference range: 160.0-380.0 µmol/L). Blood glucose, blood creatinine, glomerular filtration rate, and potassium levels were normal. Thyroid function was also normal, and blood pressure remained consistently normal upon multiple measurements. The patient’s test results are shown in **Table 1**.

**Table 1:** Laboratory Results and Reference Ranges.

October, 2018		
Parameter	Test Results	Reference range (unit)
NA	124.5↓	137-147 (mmol/L)
	121↓	
	126.9↓	
SOSM	251 ↓	275-305 (mOsm/kg H2O)
	271 ↓	
UOSM	590.00	50-1200 (mOsm/kg H2O)
	906.00	
UNA	171.70	(mmol/L)
	182.70	
8am.ACTH	43.88	5.0-78 (ng/mL)
	49.46	
8am.COR	271.90	147.3-609.3 (nmol/L)
	319.60	
24hUFC	46.80	20.26-127.55 (ug/24hr)
TSH	1.81	0.27-4.2 (mU/L)
T4	69.11	62-164 (nmol/L)
FT4	14.15	12.0-22.0 (pmol/L)
T3	1.35	1.3-3.1 (nmol/L)
FT3	4.30	3.60-7.50 (pmol/L)
LH	13.80	2.4-14 (mIU/mL)
FSH	3.90	3.5-12.5 (mIU/mL)
E2	226.00	12.4-233 (pg/mL)
P	0.20	0.057-0.893 (ng/mL)
PRL	27.03	6.0-29.9 (ng/mL)
SHBG	81.10	32.4-128 (nmol/L)
FT	0.94	<2 (%)
GH	0.30	0.126-9.88(ng/mL)
IGF-1	124.53 ↓	137-278 (ng/mL)
AFP	0.66	<15 (ng/mL)
CEA	0.69	<3.4 (ng/mL)
CA15-3	9.37	<21(U/mL)
CA19-9	4.71	<22 (U/mL)
CA-125	5.88	<35 (U/mL)
CYFRA21-1	0.88	<3 (ng/mL)
NSE	8.09	<15 (ng/mL)

NA, Serum Sodium; BP, Blood Pressure; SOSM, Serum Osmolality; UOSM, Urine Osmolality; UNA, Urinary Sodium; ACTH, Adrenocorticotropic Hormone; COR, Cortisol; 24hUFC, 24-Hour Urinary Free Cortisol; TSH, Thyroid Stimulating Hormone; T4, Thyroxine; FT4, Free Thyroxine; T3, Triiodothyronine; FT3, Free Triiodothyronine; LH, Luteinizing Hormone; FSH, Follicle Stimulating Hormone; E2, Estradiol; P, Progesterone; PRL, Prolactin; SHBG, Sex Hormone Binding Globulin; FT, Free Testosterone; GH, Growth Hormone; IGF-1, Insulin-like Growth Factor-1; AFP, Alpha-fetoprotein; CEA, Carcinoembryonic Antigen; CA153, Serum Carbohydrate Antigen 15-3; CA19-9, Serum Carbohydrate Antigen 19-9; CA-125, Serum Carbohydrate Antigen 125; CYFRA21-1, Non-Small Cell Lung Cancer Antigen; NSE, Neuron-Specific Enolase.

↓: Decrease below the reference value.

## Diagnostic assessment

According to the guidelines (1, 3), the patient was clinically diagnosed with chronic hypotonic hyponatremia. Given the patient’s urine osmolality >100 mOsm/kg H₂O, urine sodium >30 mmol/L, normal renal and thyroid function, absence of a drug history causing hyponatremia, and normal blood pressure with no signs of volume depletion, cerebral salt wasting syndrome was not considered. Instead, the possibility of SIAD and adrenal insufficiency was evaluated. Given the patient’s MRI showed an empty sella, pituitary-adrenal related hormone tests were conducted. Two 8 AM cortisol (COR) readings were 271.90-319.60 nmol/L (reference range: 147.3-609.3), while ACTH levels were 43.88-49.46 ng/L (reference range: 5.0-78). The 24-hour urinary free cortisol (COR) was 46.8 µg/24 hr (reference range: 20.26-127.55). The AVP stimulation test showed an ACTH increase >35%, peaking at 15 minutes (57.9 ng/mL), significantly above the baseline (15.44 ng/mL). Further adrenal ultrasound was performed, and the results were completely normal. Cortisol increased by more than 25%, peaking at 60 minutes (563.7 nmol/L), significantly above the baseline (182.2 nmol/L), thereby ruling out adrenal insufficiency. The patient’s test results are shown in **Table 2**.

**Table 2:** Function Tests.

Parameter	Test Results(unit)
Water Deprivation Test: Restrict daily water intake to less than 500 ml for 3 days.	
NA (Pre-Test).	127.00(mmol/L)
NA (Post-Test)	132.00(mmol/L)
Saline Infusion Test: Administer an intravenous infusion of 2 liters of normal saline (0.9% NaCl) over 4 hours.	
NA (Pre-Test)	132.00(mmol/L)
SOSM (Pre-Test)	272.00(mOsm/Kg H2O)
NA (Post-Test)	131.20(mmol/L)
SOSM (Post-Test)	272.00(mOsm/kg H2O)
AVP stimulation test: 10 IU of AVP administered via intramuscular injection.	
ACTH(-15min)	16.82(ng/mL)
ACTH(0min)	15.44(ng/mL)
ACTH(15min)	57.90(ng/mL)
ACTH(30min)	52.13(ng/mL)
ACTH(45min)	40.57(ng/mL)
ACTH(60min)	24.45(ng/mL)
ACTH(90min)	131.41(ng/mL)
ACTH(120min)	8.54(ng/mL)
COR(-15min)	201.90 (nmol/L)
COR(0min)	182.20 (nmol/L)
COR(15min)	296.90 (nmol/L)
COR(30min)	421.20 (nmol/L)
COR(45min)	196.10 (nmol/L)
COR(60min)	563.70 (nmol/L)
COR(90min)	423.00 (nmol/L)
COR(120min)	325.10 (nmol/L)

NA, Serum Sodium; SOSM, Serum Osmolality; AVP, Arginine Vasopressin; ACTH, Adrenocorticotropic Hormone; COR, Cortisol.

When evaluating adrenal cortical function through the assessment of the renin-angiotensin-aldosterone system (RAAS), significantly decreased renin levels were observed in both the supine position (<0.05 ng/mL·h) and the upright position (0.1 ng/mL·h) (reference range: 0.05-0.79 ng/mL·h). Aldosterone levels were normal while lying down (10.91 ng/dl) and elevated while standing (27.67 ng/dl) (reference range: 9.8-27.5 ng/dl). The aldosterone-renin ratio (ARR) was markedly elevated, with values of 243.33 in the supine position and 237.89 in the upright position (reference range: <20 ng/dL/ng/mL·h). The patient’s test results are shown in **Table 3**.

**Table 3:** Comparison of laboratory indicators between 2018 and 2024.

Oct 2018			Apr 2024		
Parameter	Test Results	reference range (unit)	Parameter	Test Results	reference range (unit)
RBC	3.65	3.8-5.1 (10^12/L)	RBC	4	3.8-5.1 (10^12/L)
HGB	105.00 ↓	115-150 (g/L)	HGB	121	115-150 (g/L)
HCT	0.31 ↓	0.35-0.45 ( L/L)	HCT	0.38	0.35-0.45 ( L/L)
Alb	46.00	40.0-55.0 (g/L)	Alb	45	40.0-55.0 (g/L)
Urea	2.40 ↓	2.95-7.70 (mmol/L)	Urea	3.6	2.6-7.5 (mmol/L)
UA	114.00 ↓	160.0-380.0 (umol/L)	UA	287	155-375 (umol/L)
FBG	4.59	3.9-5.9 (mmol/L)	FBG	4.8	3.9-5.9 (mmol/L)
SCr	42.00	37.0-110.0 (umol/L)	SCr	62	48-79 (umol/L)
eGFR	132.50 ↑	56-122(mL/min/1.73m2)	eGFR	112.35	56-122 (mL/min/1.73m2)
K	4.42	3.5-5.3 (mmol/L)	K	4.07	3.5-5.3 (mmol/L)
Cl	87 ↓	99-110 mmol/L	Cl	107.4	99-110 mmol/L
	93.8 ↓			106.4	
PRA (Supine)	<0.05^‡^ ↓	0.05-0.79 (ng/mL • h)	Renin (Upright)	35.52^§^	3.8-38.8 (pg/mL)
AT-II (Supine)	50.53^‡^	28.2-52.2 (ng/L)	AT-II (Upright)	95.04^§^	49-252 (pg/mL)
ALD (Supine)	10.91^‡^	4.5-17.5 (ng/dL)	ALD (Upright)	140.4^§^	40-310 (pg/mL)
ARR (Supine)	243.33	<20 (ng/dl:ng/mL.h)	ARR(Upright)	4.32	<32
PRA (Upright)	0.1^‡^ ↓	0.93-6.56 (ng/mL • h)	Renin (Upright)	21.09^§^	4.4-46.1 (uIU/mL)
AT-II (Upright)	53.43^‡^	55.3-115.3 (ng/L)			
ALD (Upright)	27.67^‡^ ↑	9.8-27.5 (ng/dL)	ALD (Upright)	8.36^§^	3-35.3 (ng/dL)
ARR (Upright)	237.89	<20 (ng/dl/ng/mL • h)	ARR(Upright)	0.4	
B-ALP	28.03 ↑	11.4-24.6 (µg/L)	B-ALP	6.71	11.4-24.6 (µg/L)
CTX	1.11 ↑	0.299-0.573 (ng/mL)	CTX	0.18	0.299-0.573 (ng/mL)

RBC, Red Blood Cell Count; HGB, Hemoglobin; HCT, Hematocrit; Alb, Albumin; UREA, Urea; UA, Uric Acid; FBG, Fasting Blood Glucose; SCr, Serum Creatinine; eGFR, Estimated Glomerular Filtration Rate; K, Serum Potassium; PRA, Plasma Renin Activity (RIA); Renin, Renin (ECL); AT-II, Angiotensin II; ALD, Aldosterone; ARR, Aldosterone-to-Renin Ratio; ALP, Bone-Specific Alkaline Phosphatase; CTX, C-Terminal Telopeptide of Type I Collagen.

Supine, Measurement taken in the supine position; Upright, Measurement taken in the upright position.

^‡^, Radioimmunoassay (RIA); ^§^, Electrochemiluminescence (ECL); ↑, Increase above the reference value; ↓, Decrease below the reference value.

To clarify the diagnosis of SIAD, a water deprivation test and saline infusion test were performed. After restricting water intake to less than 500 mL per day for three days, the patient’s serum sodium increased from 127 to 132 mmol/L. Following the saline infusion test, pre-infusion serum sodium was 132.0 mmol/L, and serum osmolality was 272 mOsm/kg H₂O; post-infusion, serum sodium was 131.2 mmol/L, and osmolality remained at 272 mOsm/kg H₂O. The patient’s serum sodium increased after water restriction, but the saline infusion test did not correct the serum sodium, with uric acid <0.24 mmol/L and blood urea nitrogen <3.6 mmol/L. Ultimately, SIAD was diagnosed. The patient’s test results are shown in **Table 2**.

Despite the patient’s clear history of traumatic brain injury, pre-surgical hyponatremia, and persistent hyponatremia more than a year post-injury, further investigation of other potential causes of hyponatremia was conducted. Investigations revealed normal tumor markers (AFP, CEA, CA 15-3, CA19-9, CA125, CYFRA21-1, and NSE). Abdominal ultrasound, echocardiogram, breast ultrasound, thyroid ultrasound, superficial lymph node ultrasound, and chest CT were all normal. Gynecological ultrasound showed a Nabothian cyst of the cervix. Painless electronic gastroscopy indicated chronic non-atrophic gastritis, while painless electronic colonoscopy revealed melanosis coli. Bone density measurements showed L1-L4 Z score of -0.1, femoral neck Z score of -0.5, and total hip Z score of -0.8(**Table 4**). The patient had active bone turnover markers, with bone-specific alkaline phosphatase (B-ALP) at 28.03 µg/L (reference range: 11.4-24.6) and C-terminal telopeptide of type I collagen (CTX) at 1.110 ng/mL (reference range: 0.299-0.573), both elevated. However, serum protein electrophoresis, immunofixation electrophoresis, and urine light chains showed no abnormalities. PET-CT revealed no signs of malignancy throughout the body, but post-surgical changes in the left skull and softening lesions near the left temporal lobe were noted. No evidence of tumor tissue secreting AVP was found, leading to the consideration of post-traumatic SIAD. After thorough discussion with the patient, a decision was made to observe and follow up.

**Table 4:** Comparison of bone density results between 2018 and 2024.

Oct 2018			Apr 2024		
Parameter	BMD (g/cm²)	Z-Score	Parameter	BMD (g/cm²)	Z-Score
L1	0.937	-0.4	L1	1.066	0.6
L2	1.085	0.2	L2	1.045	0.7
L3	1.130	0.2	L3	1.219	0.9
L4	1.061	-0.3	L4	1.149	0.4
L1-L2	1.013	-0.1	L1-L2	1.107	0.7
L1-L3	1.056	0.0	L1-L3	1.148	0.7
L1-L4	1.057	-0.1	L1-L4	1.148	0.6
L2-L3	1.109	0.2	L2-L3	1.184	0.8
L2-L4	1.091	0.0	L2-L4	1.171	0.7
L3-L4	1.093	0.0	L3-L4	1.181	0.7
FN	0.832	-0.5	FN	0.869	-0.3
WT	0.744	-0.7	WT	0.779	-0.5
GT	0.608	-1.1	GT	0.672	-0.5
TH	0.837	-0.8	TH	0.895	-0.4

BMD, Bone Mineral Density; L1, Lumbar Spine 1; L2, Lumbar Spine 2; L3, Lumbar Spine 3; L4, Lumbar Spine 4; L1-L2, Lumbar Spine 1-2; L1-L3, Lumbar Spine 1-3; L1-L4, Lumbar Spine 1-4; L2-L3, Lumbar Spine 2-3; L2-L4, Lumbar Spine 2-4; L3-L4, Lumbar Spine 3-4; FN, Femoral Neck; WT, Wards Triangle; GT, Greater Trochanter; TH, Total Hip.

In terms of treatment, the patient reported challenges in adhering to long-term water restriction and subsequently opted for oral tolvaptan therapy as an alternative. A cautious dose of a quarter tablet (3.75 mg) of tolvaptan was prescribed, taken at 22:00. Following administration, the patient experienced insomnia throughout the night, excreted a total of 3050 mL of urine over 8 hours, and lost 3 kg in weight. This was accompanied by pronounced thirst and dehydration; however, she limited her water intake to less than 500 mL during the night, despite being advised to drink based on thirst after administration. Serum sodium and osmolality increased from 123.2 mmol/L and 259 mOsm/kg H₂O to 139.3 mmol/L and 294 mOsm/kg H₂O, respectively. The medication was discontinued, and the patient was re-advised to drink water freely. The next day, the 24-hour urine volume was only 500 mL, with serum sodium level of 134.2 mmol/L and osmolality of 281 mOsm/kg H₂O. On the third day, the serum sodium was 134.2 mmol/L, and on the fourth day, it was 129.7 mmol/L. Although the patient did not experience psychiatric symptoms or demyelination associated with rapid correction of hyponatremia, she was extremely sensitive to tolvaptan, resulting in a dose reduction to 1/8 tablet (1.875 mg) taken twice a week, with advice to drink water guided by thirst.

From October 2018 to January 2021, the patient was treated with tolvaptan at a dosage of 1.875 mg twice a week, with serum sodium levels ranging from 130.7-139.5 mmol/L. Starting in 2021, the patient independently adjusted the use of tolvaptan, taking a dose of 1.875 mg only when experiencing weakness or noticing a drop in serum sodium levels. The medication was taken 2-3 times a month, with occasional serum sodium measurements showing 132.3 mmol/L before administration and 138 mmol/L after administration. From March 5, 2022 (approximately five years post-trauma), the patient no longer experienced symptoms of fatigue or poor appetite and made the confident decision to stop taking tolvaptan. On April 1, 2022, serum sodium levels were found to be normal at 141.7 mmol/L. Since then, the patient has not resumed medication until February 2024, with multiple serum sodium tests remaining normal and no associated discomfort.

Following the normalization of serum sodium, RAAS and bone metabolic markers were re-evaluated and found to be within normal ranges. RAAS evaluations were repeated using different testing methods, and both results were found to be normal. Bone density assessments indicated significant increases in bone mineral density at both the lumbar spine and hip regions. The patient’s test results are shown in **Table 3**.

Given the spontaneous resolution of the patient’s hyponatremia and the improvement in bone metabolism markers, we have further excluded the possibility of tumor-induced SIAD. Although the patient’s MRI suggested Empty Sella Syndrome (ESS), the normal adrenal function and resolution of SIAD despite persistent ESS indicate that ESS is not the cause of SIAD. We consider this case to be a spontaneous resolution of SIAD due to traumatic brain injury. Additionally, we believe that the patient’s abnormalities in RAAS and bone metabolism are also attributable to SIAD and hyponatremia resulting from the traumatic brain injury.

## Discussion

Central nervous system diseases are relatively common causes of SIAD, particularly traumatic brain injury, hemorrhages, tumors, and sphenoid surgery. In patients with subarachnoid hemorrhage, 35% present with hyponatremia during the first week, 70% of which are associated with SIAD. For those undergoing transsphenoidal pituitary surgery, the incidence of SIAD ranges from 25% to 35% (4). There is no clear evidence to suggest that the severity of trauma correlates with the development of pituitary dysfunction. The exact pathophysiology of SIAD following the traumatic brain injury (TBI) remains unclear. Potential mechanisms include damage to osmoreceptors and volume receptors in the hypothalamus due to TBI, or a resetting of the osmoreceptors’ set point, leading to inappropriate release of AVP by the hypothalamus and posterior pituitary. Alternatively, damage to the posterior pituitary might cause a transient surge of AVP into the bloodstream, resulting in temporary SIAD, followed by recovery or the development of permanent AVP deficiency. Most cases of SIAD occur shortly after central nervous system pathology and typically resolve within weeks. Born et al. reported that 36 patients developed SIAD within three weeks following severe head injury (5). Chen et al. described four cases of SIAD after TBI, with SIAD developing within four days and resolving within 10 days in all cases (6). To date, to our knowledge, only two cases of persistent SIAD lasting several years have been published. Dick et al. reported a 32-year-old male who developed SIAD following traumatic brain injury, with hyponatremia persisting for four years and eventually improving with demeclocycline treatment (7). Voort et al. described a case of enduring SIAD after TBI that resolved spontaneously after five years (8). Based on our case and the literature, we conclude that inappropriate AVP secretion following traumatic brain injury can persist for years and may eventually resolve spontaneously. Therefore, in clinical practice, it is essential to consider the possibility of post-TBI SIAD in patients with a history of prolonged hyponatremia and prior traumatic brain injury, and clinicians should remain hopeful about the potential for spontaneous resolution.

The SIE Practice Guidelines (1) and the US guidelines (9) recommend that a urine osmolality greater than 500 mOsm/kg is a strong predictor of poor response to fluid restriction. Additionally, the SIE Practice Guidelines (1), along with the US (9) and British guidelines (10), all advocate for the use of the Furst formula (urine Na + urine K/plasma Na) (11), where a ratio greater than 1 strongly indicates a failure to respond to fluid restriction. In this case, the patient’s urine osmolality was greater than 500 mOsm/kg, and the Furst formula ratio was greater than 1, indicating poor response to fluid restriction therapy. Indeed, after undergoing fluid restriction, the patient’s blood sodium level did not normalize. Therefore, tolvaptan is a relatively good option for her, as it has now been well established as an effective treatment for symptomatic hyponatremia due to SIAD (12). Onuigbo et al. (13) reported a dramatic correction of hyponatremia at a rate of 1 mEq/dL per hour over 18 hours, following the administration of 15 mg oral tolvaptan in a 32-year-old male with normal kidney function (serum creatinine 0.76 mg/dL) after traumatic brain injury (TBI). Based on this, they strongly recommend using lower doses of tolvaptan (≤15 mg/day) in younger patients with preserved renal function to prevent life-threatening pontine demyelination. Another study (14) identified baseline serum sodium and Serum Urea Nitrogen (SUN) concentrations as independent predictors of the rapidity of sodium correction in patients treated with tolvaptan for SIAD. Patients with baseline serum sodium ≤121 mmol/L and SUN ≤10 mg/dL exhibited a mean 24-hour sodium correction of 15.4 mmol/L after taking tolvaptan, which was significantly higher compared to patients with higher baseline serum sodium or SUN concentrations. This rapid correction, defined as an increase >12 mmol/L within 24 hours, predisposes such patients to a higher risk of serious neurological complications, including osmotic demyelination syndrome (ODS). Therefore, starting tolvaptan therapy at a lower initial dose (e.g., 3.75–7.5 mg) and gradual dose adjustment is strongly recommended for high-risk patients (14). Incorporating these findings into clinical practice could enhance treatment safety, prevent overcorrection, and minimize the risk of adverse neurological outcomes. In our case, the patient demonstrated high sensitivity to tolvaptan, with a 3.75 mg oral dose causing a 16.1 mmol/L increase in serum sodium within 8 hours. Even with twice-weekly doses of 1.875 mg, the patient’s serum sodium remained close to the normal range. Therefore, it is recommended to start such high-risk patients on the lowest possible dose of tolvaptan, preferably administered during the day, with close monitoring of serum sodium levels to avoid rapid increases. Tolvaptan dosage should be adjusted gradually based on the patient’s serum sodium levels and urine output.

We also observed significantly reduced renin activity during the SIAD period, with aldosterone levels either normal or elevated. There has been a reported case of a 36-year-old male with hyponatremia caused by ectopic AVP secretion from an esthesioneuroblastoma, who had a 15-year history of SIAD (15, 16). In this patient, pre-operative renin levels were normal, while aldosterone levels were significantly elevated. After resection of the esthesioneuroblastoma, aldosterone levels normalized (15). During SIAD, water retention, hyponatremia, and excessive AVP can influence the renin-angiotensin-aldosterone system (RAAS). In SIAD, the inappropriate secretion of excessive AVP leads to increased renal water reabsorption, which relatively increases blood volume and suppresses renin release. Studies (17) have shown that in SIAD patients, fluid restriction can elevate serum sodium levels to >130 mEq/L (serum osmolality >280 mOsm/kg) while reducing urine sodium to 20 mEq per day. As serum sodium increased and body water decreased, evidenced by a 10 lb acute weight loss, measured plasma renin (2950 ng/100 mL) and aldosterone (72 ng/100 mL) rose markedly (17). However, the etiology of normal or elevated aldosterone levels despite suppressed renin activity during SIAD require further exploration. Animal studies have found that AVP can act on V1a receptors to promote adrenal aldosterone release (18). AVP and the renin-aldosterone system appear to exhibit reciprocal regulatory effects (19). Additionally, aldosterone secretion is influenced by plasma osmolality. Studies by Schneider et al. (20) and Taylor et al. (21) demonstrated that changes in plasma osmolality, independent of renin-angiotensin signaling, can directly modulate aldosterone secretion. Decreased plasma osmolality stimulates aldosterone secretion (20, 21), likely via early steps in the biosynthetic pathway (21), while increased osmolality inhibits aldosterone synthesis (20). These findings suggest that during SIAD, AVP-induced water retention leads to hypoosmolality, which may sustain or even enhance aldosterone secretion even when renin activity is suppressed. Furthermore, elevated aldosterone levels can exert negative feedback inhibition on renin release. This osmolality-driven mechanism provides a plausible explanation for the observed dissociation between renin and aldosterone levels in SIAD.

This study also found that during the SIAD period, both osteoblastic and osteoclastic metabolic markers were significantly elevated, indicating a high bone turnover state. Following the resolution of SIAD, these bone metabolic markers returned to normal levels. As the patient’s bone density was initially measured at the age of 29 and the condition resolved at 35—considered the peak bone mass age—the changes in bone density before and after the disease do not carry significant clinical implications. However, the SIAD state, characterized by inappropriate secretion of AVP and hyponatremia, does indeed affect bone metabolism. A reported case of esthesioneuroblastoma with ectopic AVP secretion causing hyponatremia revealed severe osteoporosis pre-operatively (15, 16). After tumor removal, AVP and sodium levels normalized, and a dual-energy x-ray absorptiometry scan performed 7 months post-surgery showed significant spontaneous improvement in lumbar vertebrae bone mineral density (BMD) (15). This case supports the belief that SIAD has an adverse but reversible effect on bone metabolism. In a rat model of SIAD, Verbalis et al. (22) found that severe hyponatremia (serum sodium approximately 110 mmol/L) significantly reduced bone mineral density (BMD) by approximately 30% over three months. This reduction was particularly pronounced in both cortical and trabecular bone. The observed bone loss was closely associated with an increase in osteoclast numbers and a decrease in osteoblast activity (22). *In vitro* studies have demonstrated that low sodium levels enhance osteoclast (23, 24) differentiation and activity, accelerating bone resorption, while simultaneously inducing a shift in human mesenchymal stem cells (hMSCs) (25) toward adipogenic differentiation, suppressing osteogenic differentiation and impairing bone formation, ultimately leading to reduced bone mass. Although studies (26, 27) have shown that AVP acts through the AVPR1a transmembrane G-protein-coupled receptor in osteoclasts and osteoblasts, activating the intracellular ERK signaling pathway to promote bone resorption and inhibit bone formation, evidence (28) from Tolvaptan treatment indicates that improvements in bone resorption index, as well as osteocalcin levels and NTx-creatinine ratio, are closely linked to the restoration of serum sodium levels, suggesting that hyponatremia itself, rather than AVP, plays a primary role in the pathogenesis of osteoporosis. One meta-analysis demonstrated a significant association between hyponatremia and the risk of fractures and osteoporosis, with an odds ratio (OR) of 1.99 (95% CI: 1.50–2.63) in studies reporting ORs, and an increased risk of fractures with a hazard ratio (HR) of 1.62 (95% CI: 1.28–2.05, P < 0.001) in studies reporting HRs (29). Another meta-analysis further showed that hyponatremia increases the odds of fractures at all sites (summary OR 2.34 [95% CI: 1.86–2.96]) and the odds of osteoporosis (summary OR 2.67 [95% CI: 2.07–3.43]) (30). These findings support the conclusion that hyponatremia is significantly associated with osteoporosis and fractures. In the esthesioneuroblastoma case (15), it is also noteworthy that plasma aldosterone levels normalized after tumor removal. It is plausible that aldosterone levels may have influenced BMD, as hyperaldosteronism has been shown to be associated with bone loss in rat models (31). The review of the literature indicates that SIAD can adversely affect the bone metabolism, potentially increasing the risk of osteoporosis.

This study comprehensively explores the long-term trajectory of SIAD caused by traumatic brain injury, providing novel insights into the spontaneous resolution of the condition. It highlights the impact of SIAD on the RAAS and bone metabolism markers and, through the incorporation of relevant literature, discusses the underlying mechanisms, including the role of hypoosmolality in aldosterone regulation and hyponatremia in bone turnover. These findings offer valuable implications for clinical management strategies. However, this study has certain limitations. Firstly, the patient did not undergo routine health check-ups, and only serum sodium data from eight years ago are available. If pre-trauma serum sodium levels had been normal, the diagnosis of hyponatremia could have been more promptly linked to the traumatic brain injury. Secondly, after January 2022, the patient did not follow up with regular serum sodium measurements or adjust the tolvaptan treatment under medical supervision. Instead, the medication was used based on symptomatology, indicating a lapse in patient management after discharge.

## Conclusion

This report presents a rare case of persistent SIAD resulting from traumatic brain injury, which resolved spontaneously after five years. The patient demonstrated an unusual sensitivity to tolvaptan, with concurrent findings of decreased renin, elevated aldosterone levels, and active bone turnover markers. Following the five-year period, the condition resolved on its own, and subsequent evaluations revealed normalization of the RAAS and bone turnover markers. A review of the literature indicates that SIAD can impact the RAAS and bone metabolism, potentially increasing the risk of osteoporosis. For patients with prolonged hyponatremia and a documented history of TBI, it is essential to consider the possibility of post-traumatic SIAD while remaining optimistic about the potential for natural resolution. When prescribing tolvaptan, it is crucial to start with a low dose and adjust the treatment based on changes in the patient’s serum sodium levels and urine output.

## Data Availability

The original contributions presented in the study are included in the article/supplementary material. Further inquiries can be directed to the corresponding authors.
